# The Influence of Fiber Dispersion on the Properties of MgO Concrete and Engineering Applications

**DOI:** 10.3390/ma18020261

**Published:** 2025-01-09

**Authors:** Feifei Jiang, Wencong Deng, Qi Wang, Zihan Gu, Jialei Wang

**Affiliations:** 1 School of Civil Engineering, Nantong Institute of Technology, Nantong 226000, China; 999620140019@just.edu.cn; 2 China State Construction Engineering (Macau) Co., Ltd., Macau 999078, China; dengwencong@hotmail.com (W.D.); wangqi_2019@cohl.com (Q.W.)

**Keywords:** MgO expansion agent, fiber, fiber distribution, crack resistance, compressive strength

## Abstract

Adding expansion agents to compensate for concrete shrinkage is a common crack resistance technique, but excessive expansion can also increase the porosity of concrete and reduce its strength. The addition of fibers can reduce expansion and improve the compactness of concrete. However, too little fiber will not be effective in inhibition, while too much fiber will cause aggregation. In this study, steel fiber and MgO expansive agent were used at the same time, and the effect of fiber on the mechanical properties of MgO concrete was studied. The results showed that the appropriate amount of MgO (8%) could compensate for the shrinkage of concrete and slightly improve the strength of concrete. When the content reached 10%, MgO produced excessive expansion under free conditions, which reduced the strength of the concrete. After using MgO and steel fiber at the same time, steel fiber could restrain the expansion of MgO, improve the compactness of concrete, produce a “super superposition” benefit, and increase the strength of concrete by 20%. In addition, the reinforcing effect of steel fiber on MgO was closely related to its distribution. In the composite system, steel fiber not only played a “bridge role” but also needed steel fiber to effectively restrain the expansion of MgO and produce self-stress. Only when the steel fibers were evenly distributed could reliable bonding be ensured between the fibers and the matrix, and at this time, the fibers could restrain the expansion of MgO. Considering the uniformity of steel fiber distribution and construction cost, adding 8% MgO and 1% steel fiber has the maximum benefit.

## 1. Introduction

In modern engineering construction, concrete is the most commonly used building material. But at the same time, the problem of cracking in the hardening and service process of concrete is becoming increasingly prominent. The emergence of cracks not only affects the aesthetics of the structure but also may lead to a decrease in the durability of the concrete, which in turn affects the overall safety and service life of the engineering structure [1,2,3]. The main causes of concrete cracking include factors such as drying shrinkage, autogenous shrinkage, temperature stress, and external environmental changes [4,5,6]. Especially in modern high-strength concrete, due to the low water–cement ratio, high strength, and concentrated hydration heat, drying shrinkage and temperature shrinkage are more pronounced [7,8]. These shrinkages generate tensile stress inside the concrete, ultimately leading to cracks [9,10].

Adding an expansion agent is a commonly used method for crack resistance. The hydration of the expansion agent produces expansion, which can counteract the drying shrinkage and self-shrinkage during the early hardening process of concrete and reduce the risk of cracks caused by shrinkage stress. Traditional expansion agents, such as sulphoaluminate and CaO expansion agents, can provide some expansion compensation. However, due to their fast reaction speed, large early expansion, and small expansion in the middle and late stages, they cannot match the long-term shrinkage process of concrete [11,12,13]. On the other hand, a MgO expansion agent has become a more ideal compensating shrinkage material due to designable expansion [14], low water demand [15], and stable hydration products [16], especially for structures requiring durability and crack resistance, such as underground engineering, bridges, and water conservancy projects [17].

However, some people doubt that the MgO expansive agent will reduce the compressive strength of concrete. They believe that MgO hydration will not only compensate for the shrinkage and produce macro expansion but also increase the porosity of concrete, which will have an adverse impact on the strength. Wang [18] found that after adding 8% MgO, the expansion of MgO increased the porosity of concrete and reduced the compressive strength of 3d, 28d, and 180d by 17.2%, 16.4%, and 14.6%, respectively. What is more serious is that the excessive expansion will produce harmful expansion inside the concrete, damaging the microstructure and integrity of the concrete. Ye [19] found that adding 10% MgO resulted in an excessive expansion of MgO under high-temperature conditions, leading to expansion cracks and a 37% decrease in the compressive strength of concrete.

**Figure 1:**
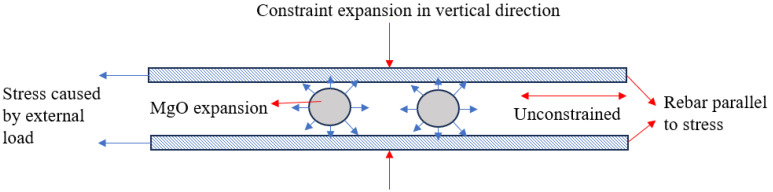
Expansion of MgO in reinforced concrete structures.

In order to avoid excessive expansion of MgO causing a decrease in concrete strength, researchers restricted the expansion of MgO concrete by applying constraints. Zhou [20] studied the application of MgO in concrete-filled steel tubes and found that the compensation of MgO compensated for the shrinkage of concrete avoided the void between the inner wall and concrete. In addition, the steel tube restrained the expansion and improved the density of the concrete, thereby increasing the bearing capacity by 19%. However, the steel tube–concrete composite structure is not common in engineering. Therefore, researchers studied common reinforced concrete structures, where steel bars also restrain the expansion of concrete. Yu [21] found that in the reinforced concrete structure, the longitudinal reinforcement restrained the expansion of MgO, improved the compactness of concrete, reduced the 28-day porosity by 6.8%, and improved the strength of concrete. However, as shown in Figure 1, because the longitudinal stressed reinforcement is distributed in the direction of the principal stress generated by the external load, it can effectively restrain the expansion in the vertical direction, but there is no constraint in the parallel direction, so the constraint effect is limited.

Based on the above analysis, the performance of MgO concrete can be improved by the constraint, but both the steel tube and the stressed reinforcement have certain limitations. In the previous study, our group attempted to use steel fibers to constrain the expansion of MgO. The randomly distributed steel fibers can constrain the expansion in any direction, thereby enhancing the strength of the concrete [22]. However, it has also been found that the distribution of fiber has a great influence on the performance of concrete. When the fibers are unevenly distributed and agglomerated, the expansion cannot be limited, and the “strengthening and toughening effect” cannot be played. Previous studies have shown that the distribution of steel fibers has a significant impact on the mechanical and durability properties of concrete. When the fibers are unevenly distributed, or oriented perpendicular to the principal tensile stress, the reinforcement and toughening effects of the fibers and the effect of restraining expansion are greatly reduced [23,24]. In the composite system, the distribution of steel fibers will not only affect its “bridging effect” but also affect its constraint effect on MgO expansion. Therefore, the study of fiber dispersion is more valuable for fiber—MgO composite systems than ordinary fiber concrete.

This paper takes the super high-rise buildings in Macao as the research object and analyzes in detail the influence of the coupling effect of MgO and fiber on the performance of mass platform concrete. The basement platform of high-rise buildings usually has a large area and thickness. After concrete is poured, there will be significant temperature drop shrinkage and drying shrinkage, which can easily lead to shrinkage cracks. Therefore, the MgO expansion agent is used to compensate for shrinkage, and steel fibers are added to further improve the strength and crack resistance of concrete. The internal mechanism of the influence of fiber dispersion on the performance of MgO concrete is studied in detail through the layered cutting experiment. Finally, the optimal dosage of steel fibers is confirmed, and fiber-reinforced MgO concrete is successfully applied in engineering construction. The research findings are of significant importance in addressing the issue of MgO reducing the strength of concrete.

## 2. Materials and Methods

### 2.1. Materials

The cement is PO 52.5 grade ordinary Portland cement, with a specific surface area of 348 m^2^/kg and a specific density of 3142 kg/m^3^. The fly ash is Class I fly ash with a specific surface area of 409 m^2^/kg and a sulfate content of 1.2%. The detailed chemical composition of cement and fly ash is shown in Table 1. The fine aggregate is river sand, with an apparent density of 2623 kg/m^3^ and a fineness modulus of 2.93. Coarse aggregate is 5–20 mm limestone, with an apparent density of 2728 kg/m^3^ and a bulk density of 1478 kg/m^3^. The steel fiber is a wavy steel fiber with a tensile strength of 520 MPa, a density of 7.8 kg/m^3^, a diameter of 0.58 mm, and a length of 38 mm. The activity of the MgO expansion agent is 105 s. The water reducer is a polycarboxylic acid high-performance water reducer with a water reduction rate of 28.1%. To meet the construction and workability of pumping during later on-site construction, the amount of water-reducing agent is adjusted to ensure that the concrete slump of each mixing ratio is always within a reasonable range (100–140 mm) in the test. MgO is added in three different concentrations: 6%, 8%, and 10% by mass. The steel fiber is set at three different concentrations: 0.5%, 1%, and 1.5% by volume. Detailed mix proportions of concrete are shown in Table 2. In order to make the fiber distribution as uniform as possible, the mixing process of secondary feeding is adopted. First, put the coarse and fine aggregates, cement and fly ash into the mixer and mix for 3 min; then, add steel fiber and mix again for 3 min. Finally, add water and water-reducing agent, and stir for another 5 min.

### 2.2. Methods

#### 2.2.1. Compressive Strength Test and Splitting Tensile Strength Test

The compressive strength test and splitting tensile strength test are conducted in accordance with the test method described in the “Test Method for Mechanical Properties of Ordinary Concrete” (GB/T 50081-2016) [25], using a specimen size of 150 mm × 150 mm × 150 mm. After demolding, each fresh concrete test block is placed in a curing room and tested for compressive strength when it reaches the corresponding testing age. The curing temperature is 20 °C ± 2 °C, and the curing humidity is above 95% RH. The loading speed is 0.5 MPa/s.

#### 2.2.2. Fiber Distribution Test Process

**Figure 2:**
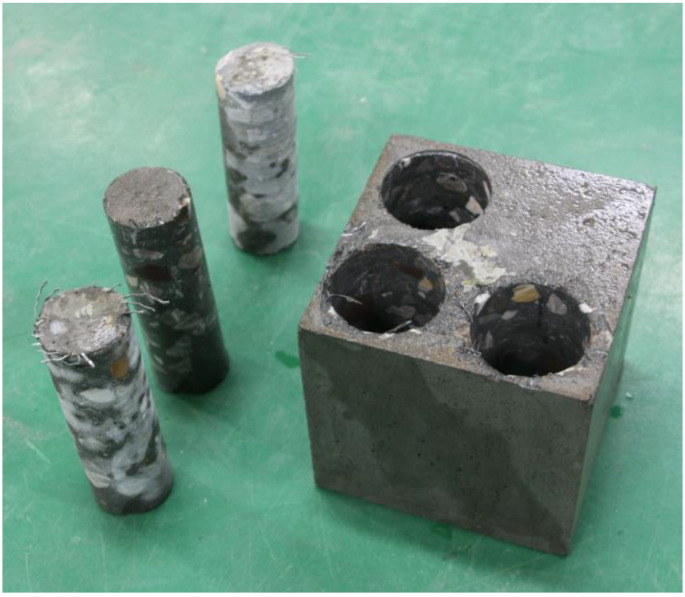
Drilling of concrete.

**Figure 3:**
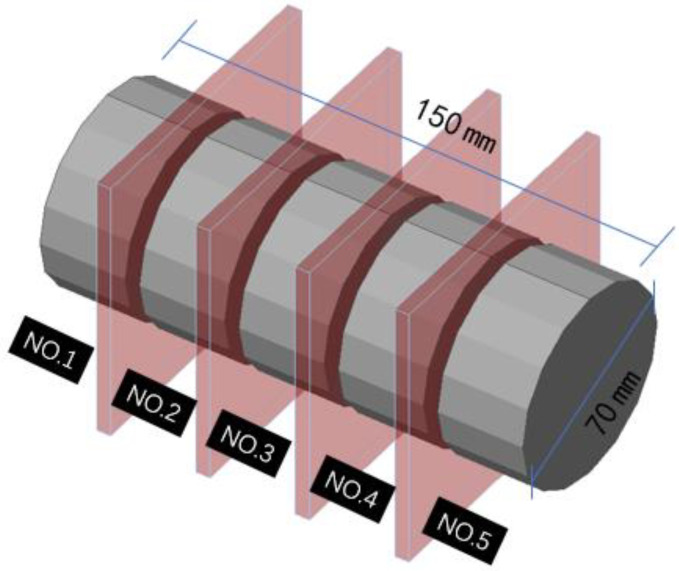
Schematic diagram of concrete cutting.

(1) Cutting of test piece. First, as shown in Figure 2, drill and core the steel fiber concrete to obtain a cylinder with a diameter of 70 mm and a height of 150 mm. Next, divide the cylinder into five equal parts along the height direction, numbered 1–5 (1 for the top surface and 5 for the bottom surface). The height of each small test block is 30 mm, and the cutting diagram is shown in Figure 3;
(2) Crushing of test pieces. Place the cut small test block flat on the workbench and slowly tap it with a hammer to break it;
(3) Weighing of steel fibers. Use a strong magnet to suck out all steel fibers from the crushed small test block and weigh them, recording the weight as mi (where i is the test piece number).

#### 2.2.3. Calculation Method

After weighing the steel fibers in each section of the test piece, the steel fiber content of the five parts from the top to the bottom of the test piece is calculated. The fiber content of each type of concrete height is taken as the average value of eight test pieces. Two methods are used to analyze the uniformity of steel fiber distribution at different locations.

(1) Direct calculation method.

Directly compare the fiber volume fraction ($x_{i}$) of specimens with different heights to evaluate the distribution. The specific calculation method is shown in Formula (1).

(1) $$x_{i}=\frac{m_{i}/\rho_{F}}{V_{i}}$$

where $x_{i}$ is the volume fraction of steel fiber in the ith small test block, $m_{Fi}$ is the mass of steel fiber in the *i*th small test block, $\rho_{F}$ is the density of steel fiber, and $V_{i}$ is the measured volume of the ith test block

(2) The fiber variation coefficient ($\psi_{\left(x\right)}$) and fiber distribution coefficient *γ* are used to evaluate the distribution of fibers. The calculation method is shown in Formulas (2) and (3).

(2) $$\psi (x)=\sqrt{\frac{\sum {\left(x_{i}/\bar{x}-1\right)}^{2}}{n}}$$

(3) $$\gamma =\exp\left[-\psi (x)\right]$$

where *n* is the number of test pieces divided, and *n* = 5 in this experiment; *x_i_* is the measured volume fraction of steel fibers in the ith small test block; $\bar{x}$ is the average value of the measured volume fraction of steel fibers in 5 small test blocks; and *γ* is the distribution coefficient of steel fibers in the test piece. When *γ* is closer to 1, it indicates that the steel fibers are more uniform. When *γ* is closer to 0, it indicates that the steel fiber is less uniform.

#### 2.2.4. Microstructure Testing

A scanning electron microscope (SU3500, Hitachi, Chiyoda, Japan) is used to scan the fiber–matrix interface transition zone to study the effects of steel fiber distribution and MgO expansion on the microstructure of concrete. In order to reduce the influence of surface laitance on the test, the sample 5 cm below the surface is selected for microstructure test.

## 3. Results and Discussion

### 3.1. Mechanical Properties of Concrete

**Figure 4:**
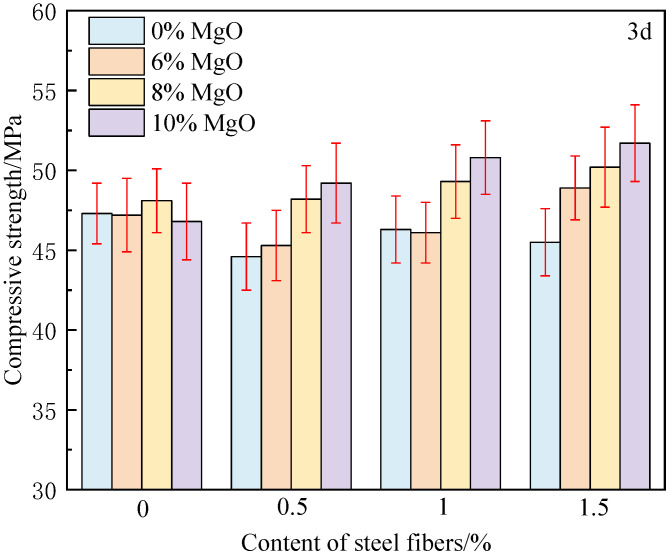
Compressive strength of concrete at 3 days.

**Figure 5:**
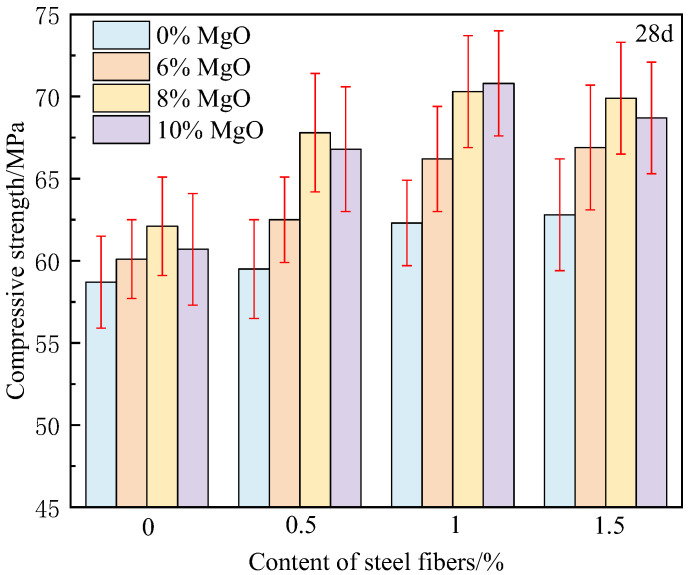
Compressive strength of concrete at 28 days.

The compressive strength of 3 days and 28 days concrete is shown in Figure 4 and Figure 5. From Figure 4, it can be found that in the early stage, the effect of steel fiber on the strength of concrete is not obvious, and even some concrete strength has decreased, such as the strength of MF-0-5 is reduced by 5.7%. There are two main reasons for this. The first is that in the early stage of concrete (within 3 days), the hydration of cement is not yet complete, and the matrix has not yet formed sufficient strength. Steel fibers come into contact with an incompletely hydrated matrix, resulting in low interfacial bonding strength. The second reason is due to the shrinkage of the cement matrix. After the cement is hardened, drying shrinkage and chemical shrinkage occur, and tiny gaps and internal tensile stresses are generated at the interface between the steel fibers and the matrix, weakening the fiber–matrix bond and forming microstructural defects, resulting in a decrease in compressive strength. This results in M-10-0.5 having a strength of 4.8 MPa higher than MF-0-0.5. The addition of MgO compensates for shrinkage, resulting in higher strength at equal fiber content.

On the other hand, when there is no constraint (no steel fiber added), the harmful expansion occurs when the MgO content reaches 10%. The strength of M-10-0 is 1.4 MPa lower than that of M-8-0, indicating that in engineering, the amount of MgO cannot be increased blindly. Under free conditions, excessive MgO can cause harmful expansion, increase the porosity of concrete, reduce its strength, and even lead to expansion cracks. This is also the main reason why structural designers currently oppose the use of MgO in engineering construction.

In order to further analyze the influence of the composite effect of MgO and steel fibers on compressive strength, the compressive strength enhancement coefficient *μ_c_* of concrete under various mix proportions was calculated according to Formula (4), and the results are shown in Table 3 and Table 4.

(4) $$\mu_{c}=\frac{f_{ci}-f_{co}}{f_{co}}\times 100\%$$

where *f_ci_* is the compressive strength of fiber-reinforced expansive concrete with different dosages, and *f_co_* is the compressive strength of ordinary concrete.

From Table 4, it can be observed that after 28 days, the hydration reaction of cement is basically completed, and the strength of the concrete matrix significantly increases. After hydration, the matrix becomes denser, and the strength of the interface transition zone between steel fibers and the matrix is enhanced, which helps to fully utilize the reinforcing effect of steel fibers. Therefore, after adding steel fibers, the 28 days reinforcement coefficient is greater than that of 3 days. When the steel fiber content is 0.5%, the strength is only 1.4% higher than that of M-0-0. This is mainly due to insufficient fiber content and uneven distribution, which can not give full play to the “bridge” role of fibers. When the dosage increases from 0.5% to 1.0%, the reinforcement coefficient rapidly increases from 1.4% to 6.1%, mainly due to the uniform distribution. When the dosage continues to increase from 1.0% to 1.5%, the reinforcement coefficient only increases from 6.1% to 7.0%. This is mainly because although the fiber dosage increases, the fiber uniformity deteriorates, and even agglomeration occurs. Fiber aggregation leads to a significant decrease in the bonding strength between the matrix and fibers, and the residual strength of the concrete decreases after the matrix cracks, making it impossible to fully utilize the reinforcing effect of fibers. Considering the high price of steel fiber, it is recommended that the dosage of steel fiber should not exceed 1% in engineering applications.

On the other hand, after using both MgO and steel fibers simultaneously, the strength of concrete increased by 20.6%, which is greater than the sum of the increments when using MgO and steel fibers alone (7.0% + 5.8%), indicating that MgO and steel fibers have produced a super superposition effect. MgO expansion agent produces an expansion effect during the hydration process. When the expansion is limited by steel fibers, compressive stress (self-stress) is generated between the fibers and the matrix. This self-stress effectively counteracts the shrinkage tensile stress in the early stage of concrete hardening, avoiding the generation of microcracks caused by shrinkage. The pre-stress applied by the MgO expansion agent to the matrix can generate a high bonding force in the interface transition zone, making the interface bonding between steel fibers and cement matrix more stable, reducing the occurrence of interface microcracks, and thus better exerting the reinforcement effect of steel fibers. Of course, this strengthening effect is closely related to the distribution of fibers, and only a uniform distribution of fibers can better constrain the expansion of MgO.

**Figure 6:**
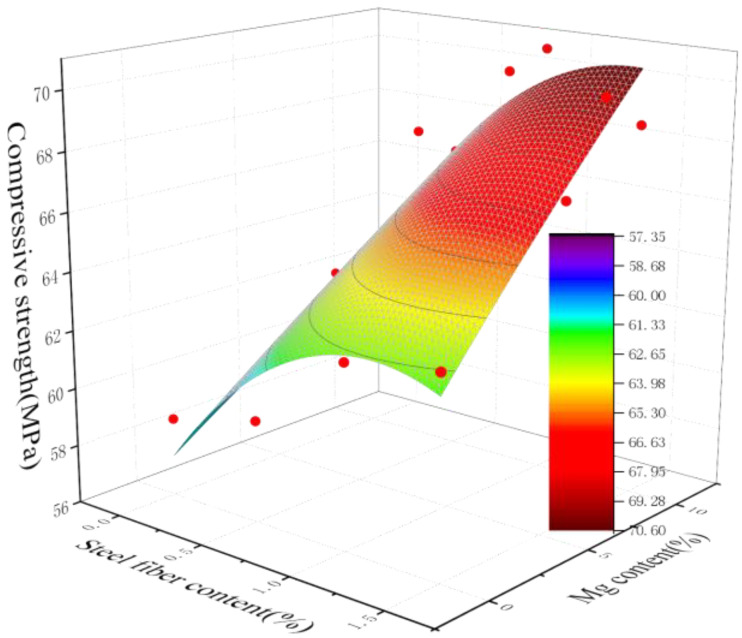
Regression analysis of the compressive strength of fiber-restrained expansive concrete.

From the previous analysis, it can be seen that the dosage of steel fibers and MgO expansion agent can both affect the compressive strength of concrete. By conducting regression analysis using Origin analysis software 2022 on the strength of different dosages, a formula for predicting the compressive strength of concrete under the dual factor effect is obtained to quantitatively calculate the influence of both on the strength of concrete. This provides a calculation method for predicting the strength of fiber-reinforced MgO concrete in engineering design. The prediction formula is calculated based on two raw materials—MgO with an activity of 105 s and steel fiber with a length of 38 mm—used in this experiment. If the raw materials change, the parameters of the model can be corrected through a small amount of supplementary experiments to improve the prediction accuracy. The fitting results are shown in Figure 6, and the calculation method is shown in Formula (5).

*f_c_* = −4.08*F*^2^ − 0.08*M*^2^ + 9.19*F* + 0.54*M* + 0.26*MF* + 57.4 (5)

where *f_c_* is the compressive strength of concrete at 28 days, *M* is the content of MgO, and *F* is the content of steel fiber.

**Figure 7:**
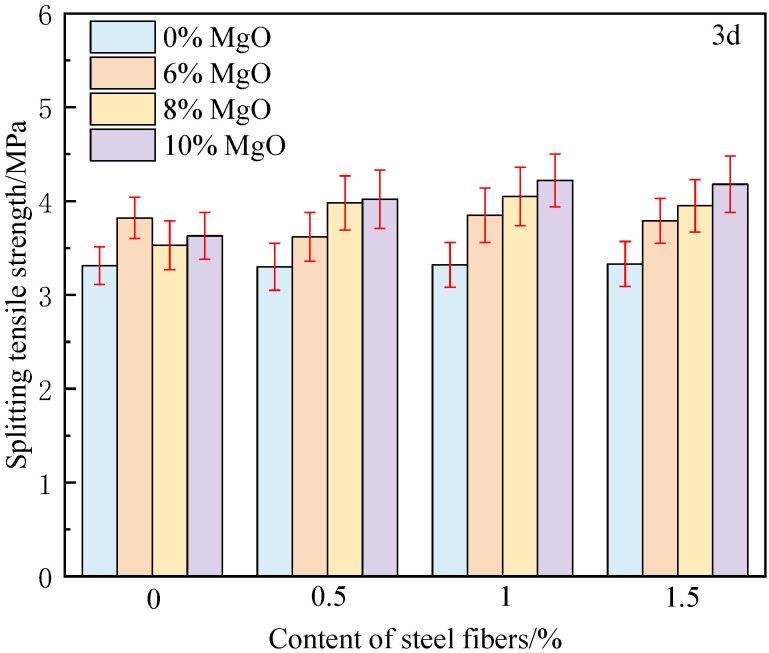
Splitting tensile strength of concrete at 3 days.

**Figure 8:**
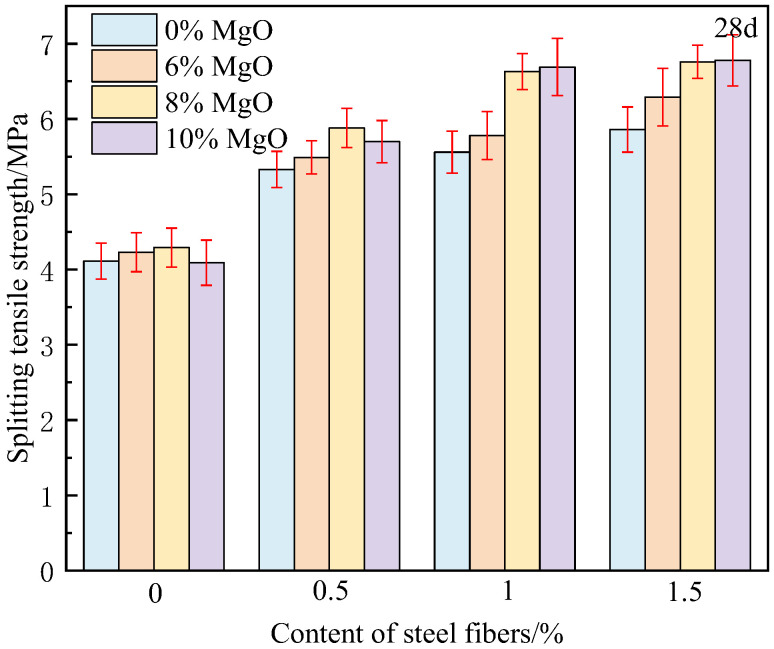
Splitting tensile strength of concrete at 28 days.

Figure 7 and Figure 8 show the splitting tensile strength of concrete at 3 days and 28 days, respectively. It can be found that when steel fibers and MgO expansion agent are used in combination, MgO further stimulates the ability of steel fibers, and the tensile strength of concrete is improved by 33.6–65.0%. When the MgO content is 8%, the tensile strength of 1% and 1.5% steel fiber-reinforced concrete reaches 6.63 MPa and 6.76 MPa, respectively. Compared to ordinary concrete, it has increased by 61.3% and 64.5%, respectively, which is much higher than the sum of the increments when used separately. Moreover, the composite effect enhances the splitting tensile strength more than the compressive strength. This is mainly due to the transfer of load to adjacent steel fibers after the matrix cracks. Due to the composite effect, the bond strength between the matrix and fibers is improved, which enhances the residual strength of the concrete after cracking and allows it to continue to withstand tensile stress.

### 3.2. Analysis of Steel Fiber Distribution at Different Depths

The changes in fiber volume fraction of concrete slices with different MgO contents at different depths are shown in Table 5, Table 6, Table 7 and Table 8. Based on the measured volume fractions of each slice, the variation coefficient (*ψ*) and distribution coefficient (*γ*) can be calculated.

**Figure 9:**
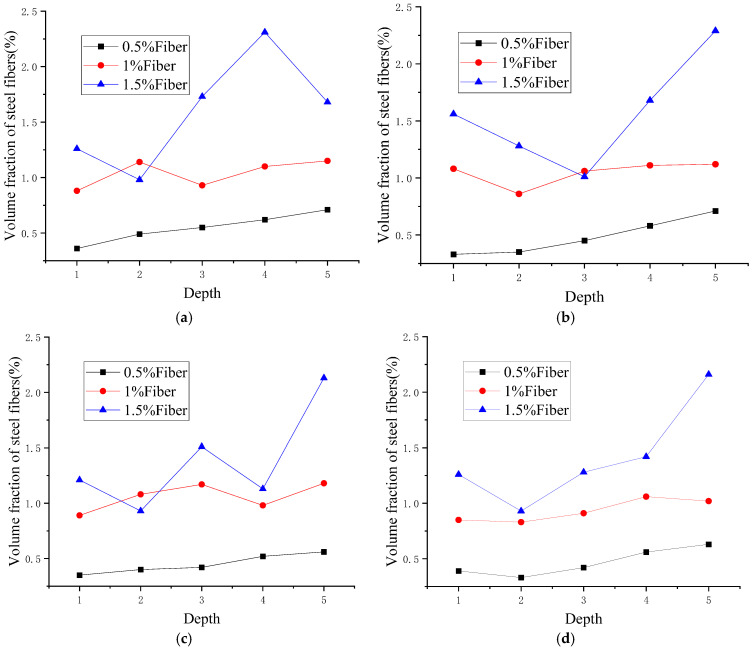
Changes in steel fiber content at different depths: (**a**) 0% MgO; (**b**) 6% MgO; (**c**) 8% MgO; (**d**) 10% MgO.

**Figure 10:**
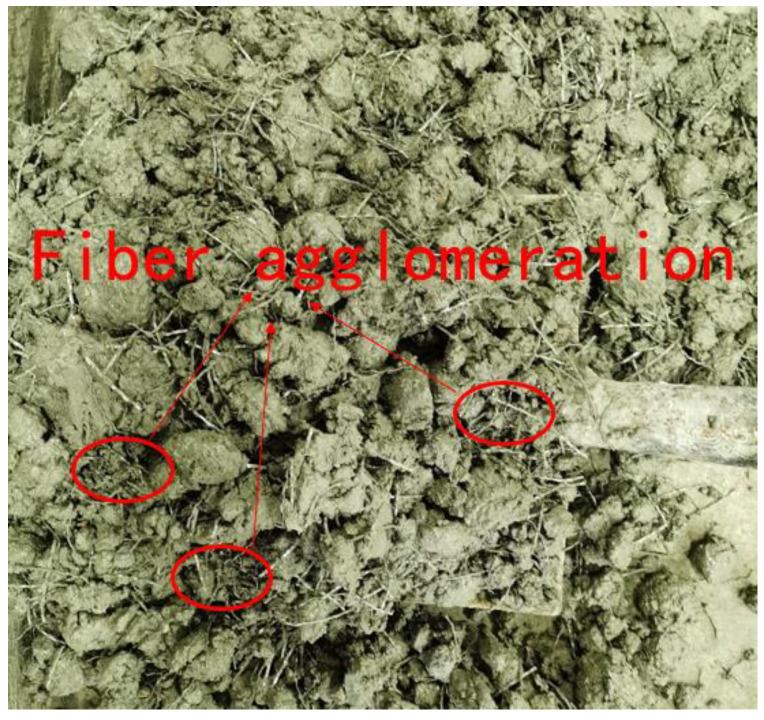
Morphology of concrete with 10% steel fiber content.

The variation of the steel fiber volume fraction in samples of different heights is shown in Figure 9. From Figure 9, it can be observed that the volume fraction of steel fibers in each section does not remain constant but varies along the height direction. When the steel fiber content is small (0.5%), gravity plays a dominant role. As the depth increases, the volume content of steel fibers shows a significant increase trend, rapidly increasing from 0.36% to 0.71%. This drastic change in content along the depth position can easily lead to local defects in the cement matrix. When the steel fiber content increases to 1%, due to the mutual friction between steel fibers and the obstruction of coarse aggregates, the fiber settlement caused by gravity is no longer significant, and the fiber content is evenly distributed along the height direction. When the steel fiber content continues to increase to 1.5%, as shown in Figure 10, there is a significant agglomeration of steel fibers. The steel fiber content varies greatly at different heights, with a maximum of 2.31% and a minimum of only 0.98%. This also explains why the strength of concrete decreases instead of increasing when the steel fiber content increases to 1.5%. After fiber agglomeration, the following three adverse consequences will occur: (1) Fiber agglomeration reduces the uniformity of concrete, reduces the bond strength between fiber and matrix, and the weak interface between fiber and matrix will become a channel for harmful ions to penetrate, thus reducing the durability of concrete. (2) Agglomeration reduces the constraint of fiber on MgO expansion and reduces self-stress, which is unfavorable to crack resistance. (3) Due to the increase in fiber content, it also increases the construction cost, resulting in a waste of resources. On the other hand, only after the fibers are evenly distributed can a reliable connection between the fibers and the matrix be ensured. After the matrix cracks under applied load, the fibers can continue to bear tensile stress and avoid crack propagation. This can improve the residual strength of concrete after cracking. Moreover, reducing the number and width of cracks can effectively decrease the penetration rate of harmful ions, which has a high value in improving the durability of concrete. Meanwhile, uniform fiber distribution can better constrain the expansion of MgO, generate significant self-stress, and improve the volume stability of concrete.

**Figure 11:**
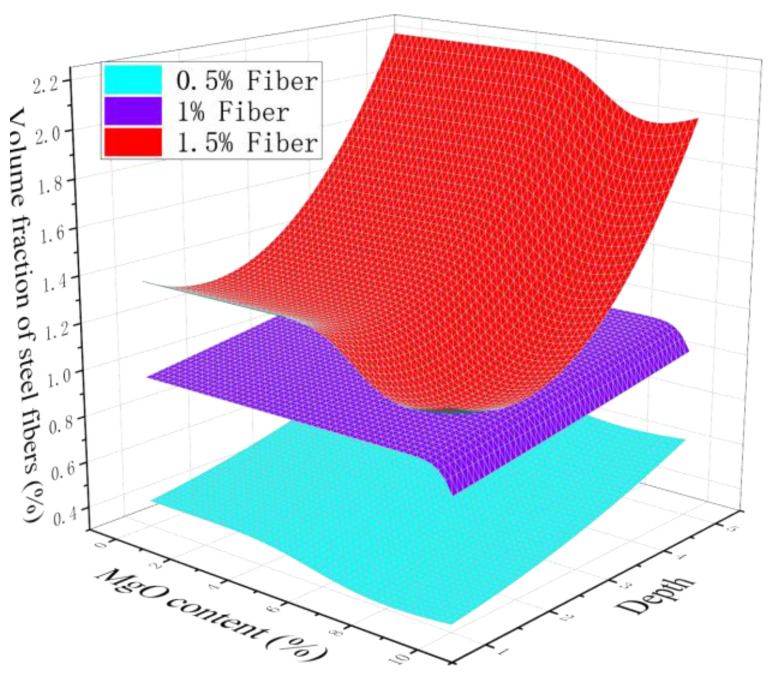
Fitting of volume fraction of steel fibers with different depths and fibers.

Figure 11 shows the fitting surface of the volume fraction of steel fibers with different dosages. For specimens with different steel fiber dosages, there are significant differences in the fiber volume fraction. When the steel fiber content is 1%, the volume fraction of fibers is most stable and is minimally affected by depth and MgO content. When the steel fiber content is 0.5%, the stability of the fiber volume fraction slightly decreases and is less affected by the MgO content but more affected by depth. When the content of steel fiber is 1.5%, the stability of the fiber volume fraction is poor, and the fiber agglomerates and fluctuates violently with depth.

#### Distribution Coefficient of Steel Fibers

**Figure 12:**
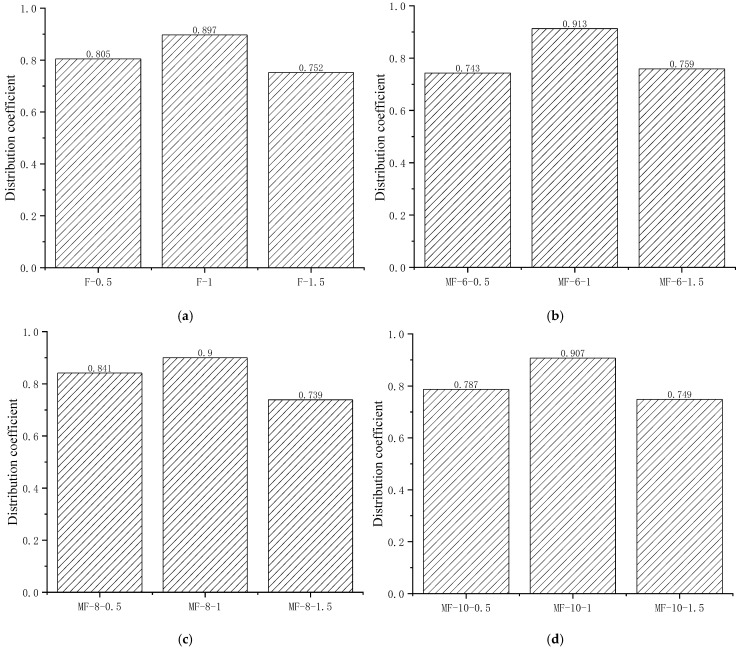
Distribution coefficient of steel fibers in concrete: (**a**) 0% MgO; (**b**) 6% MgO; (**c**) 8% MgO; (**d**) 10% MgO.

The fiber distribution coefficients of different steel fibers and MgO content are shown in Figure 12. From Figure 12, it can be observed that for concrete specimens without MgO, as the fiber content increases from 0.5% to 1%, the distribution coefficient increases from 0.81 to 0.90, and the fiber distribution gradually becomes more uniform. When the fiber content increases from 1% to 1.5%, the distribution coefficient rapidly decreases to 0.75, causing strong agglomeration of fibers and seriously affecting the uniformity of distribution. Due to the consumption of water during the hydration of MgO, theoretically, as the dosage of the MgO expansion agent gradually increases, the flowability of concrete greatly decreases, and the viscosity also increases, which will have a certain impact on the distribution of fibers. However, in this experiment, in order to ensure the pumpability of concrete construction, the amount of water-reducing agent was increased to ensure the construction performance of concrete. Therefore, the MgO expansion agent has a relatively small impact on fiber dispersion. Overall, when the steel fiber content is 1%, the volume fraction of fibers is the most stable and is minimally affected by depth and MgO content. When the steel fiber content is 0.5%, the stability of the fiber volume fraction slightly decreases and is less affected by the MgO content but more affected by depth. The steel fiber content is 1.5%, and the stability of the fiber volume fraction is poor. Due to random fiber aggregation, the content of steel fibers fluctuates violently. Therefore, in terms of fiber distribution, 1% of steel fibers have the most uniform spatial distribution, which can effectively enhance the reinforcing effect of steel fibers and also have a stronger restraining effect on MgO expansion.

### 3.3. Microstructure of Concrete

**Figure 13:**
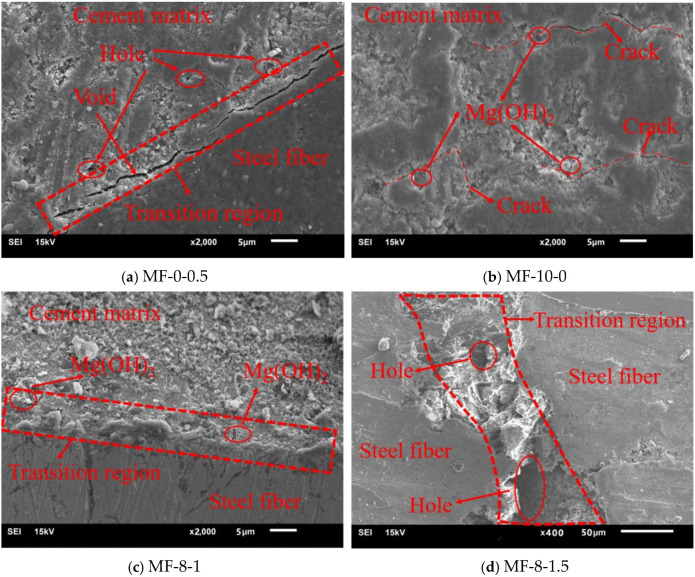
Microstructure of concrete.

As shown in Figure 13a, when steel fibers are used alone, due to the hydrophobicity of steel fibers and the “sidewall benefit”, the water/binder ratio on the fiber surface is greater than that of the matrix [26], resulting in some holes in the transition zone. On the other hand, due to the continuous hydration and shrinkage of cement after concrete pouring, there is a gap between the fiber and the matrix. The existence of holes and gaps reduces the strength of concrete.

It can be seen from Figure 13b that when MgO is used alone and the content is large, reaching 10%, a large number of expansion cracks are generated in the matrix. From the energy spectrum, it can be found that there is a large amount of Mg (OH) _2_ in the cracks, indicating that this is the harmful expansion caused by excessive MgO. The relevant literature [18] indicates that MgO expansion increases the porosity of concrete, thereby reducing its compactness. As a result, the 28-day compressive strength of the concrete decreases by 16.4%. Therefore, when using MgO to compensate for concrete shrinkage, attention should be paid to avoid the negative effect of excessive expansion on strength and durability.

It can be found from Figure 13c that when 8% MgO and 1% steel fiber are reasonably added, the steel fiber is evenly distributed without agglomeration. And the steel fiber can restrain the expansion of MgO from producing self-stress, which improves the compactness of the fiber–matrix interface transition zone and greatly improves the “bridge effect” and “expansion limiting effect” of the fiber.

As shown in Figure 13d, when the steel fiber content reaches 1.5%, the fibers are locally agglomerated, further increasing the water–binder ratio in the fiber transition zone. After the matrix hardens, the excess water dries, resulting in a large number of holes, which seriously reduces the fiber–matrix bond strength and weakens the role of steel fibers.

### 3.4. Engineering Application

#### 3.4.1. Project Overview

This construction project is a super high-rise building in Macao, with a total of six residential buildings of 149.90 m, 50 floors high, and one basement. The project covers an area of about 20,000 square meters and a construction area of about 330,000 square meters. The foundation structure mainly uses bored piles and pre-stressed concrete piles, which are connected to each pile cap by a base plate. The basement in this project is 267.9 m long and 139.7 m wide.

#### 3.4.2. Analysis of the Performance of the Concrete in the Basement Platform

(1) Huge size: In super high-rise buildings, the concrete platform has a large surface area and substantial thickness. The large surface area leads to rapid drying shrinkage of concrete, resulting in significant drying shrinkage. And the substantial thickness results in the slow dissipation and accumulation of heat generated by the hydration reaction, leading to a significant temperature drop and shrinkage at later stages. When the shrinkage is restrained, tensile stress develops, which can cause cracking in the concrete;
(2) Complex load: The platform needs to withstand the combined effects of static loads from the upper structure and dynamic loads from vehicles. Therefore, it requires concrete to have high strength;
(3) High waterproof requirements: The basement floor concrete, as the bottom structure of the building, needs to have good waterproof performance to prevent damage to the building caused by groundwater leakage;
(4) Durability requirements: Due to the long service life of super high-rise buildings, the concrete of the platform needs to have good durability and crack resistance to cope with long-term loads and environmental factors.

**Figure 14:**
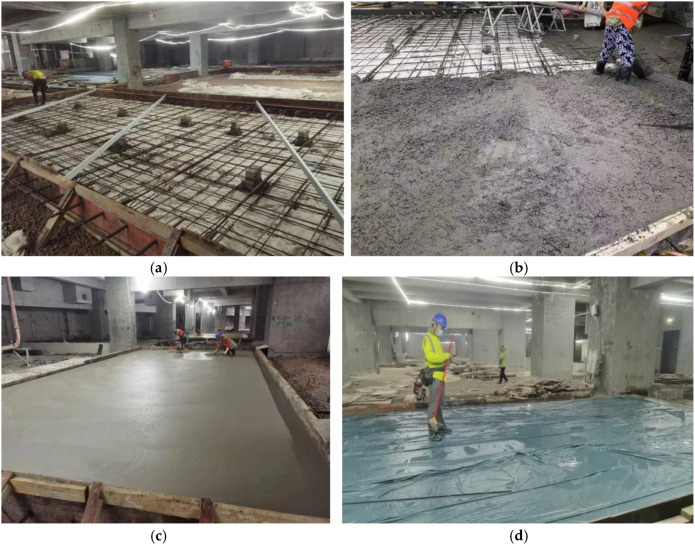
Platform concrete construction: (**a**) Reinforcement binding; (**b**) concrete pouring; (**c**) surface finishing; (**d**) maintenance.

Through the above analysis, it is found that the platform concrete has high requirements for crack resistance, strength, and durability, so 8% MgO and 1% steel fiber are added during construction. Detailed construction coordination is shown in Table 2. The main construction process includes cushion construction, steel bar binding, concrete pouring, surface finishing, and concrete curing, as shown in Figure 14.

#### 3.4.3. On-Site Testing

**Figure 15:**
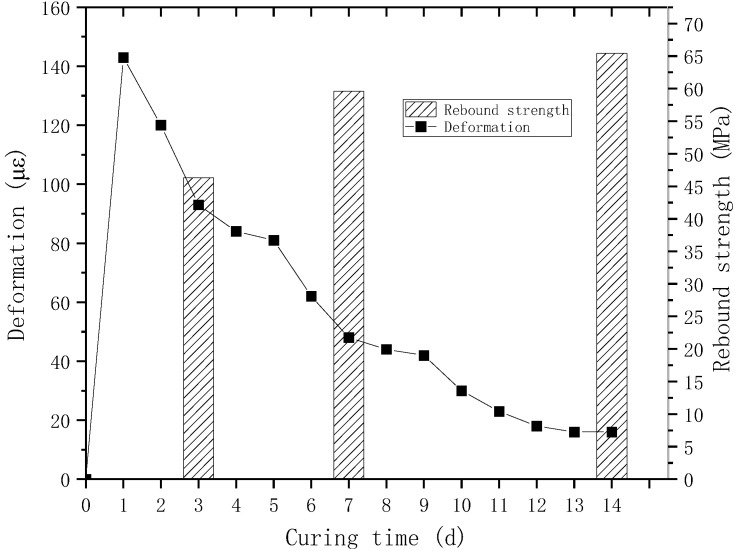
On-site measured deformation and strength of concrete.

To avoid the accumulation of hydration heat in the concrete and reduce the accumulation of shrinkage deformation, the concrete is poured into separate sections. After the curing reaches the specified age, the strength of the concrete is tested using the rebound method, and its deformation is measured using strain gauges. The detailed results are shown in Figure 15. From Figure 15, it can be found that in the early stage, due to the heat release of cement hydration, the concrete undergoes thermal expansion, reaching 143 με at 1d. After that, as the temperature decreases, the volume shrinks. At this time, the hydration expansion of MgO compensates for the temperature drop shrinkage and drying shrinkage, and the concrete remains in a slightly expanded state during the testing period of 14 days without experiencing shrinkage cracking problems. And the strength also exceeds the design strength (50 MPa). Therefore, the use of MgO and steel fiber composite can meet the construction requirements.

#### 3.4.4. Economic Analysis

(1) Initial material cost

For every cubic meter of concrete, 8% MgO (by mass) and 1% steel fiber (by volume) are added, equivalent to 40 kg of MgO and 78 kg of steel fibers. The unit price of MgO is 1 RMB/kg, and the unit price of steel fiber is 3 RMB/kg. Therefore, the additional composite material cost per cubic meter of concrete is 40 × 1 + 78 × 3 = 274 RMB.

(2) Post-Maintenance cost analysis

  (a) Crack resistance

MgO provides a micro-expansion effect that offsets shrinkage stress, significantly reducing the risk of concrete cracking. In the long term, fewer cracks help prevent waterproof layer failures and leakage risks, saving substantial costs on crack repairs. Steel fibers enhance the toughness and tensile strength of concrete, which is especially effective in preventing the propagation of cracks, thereby reducing costs associated with repairing structural damage caused by cracks. When they are used simultaneously, steel fibers constrain the self-stress generated after MgO expansion, further improving the concrete’s strength and crack resistance.

  (b) Durability

The combined use of MgO and steel fibers improves the density of concrete, enhancing its impermeability. For underground structures, the improved waterproofing and corrosion resistance reduces the long-term repair costs caused by environmental degradation.

(3) Economic comparison

The maintenance cost is calculated based on the regular maintenance frequency of the super high-rise basement platform. The cost of repairing cracks in ordinary concrete is 100 RMB per square meter per year. After using MgO expansive agent and steel fiber, the cracking probability of concrete can be reduced by more than 50%, and the later repair cost can be reduced to 50 RMB per square meter or even lower. Using a platform area of 10,000 square meters as an example, the maintenance cost for 10 years can be calculated. The repair cost for cracks in ordinary concrete is RMB 1 million, while the repair cost for composite material concrete is only RMB 500,000.

(4) Overall recommendations

The simultaneous use of MgO expansion agents and steel fiber can significantly reduce crack-related maintenance costs and water seepage losses, especially for complex underground structural environments, with significant economic benefits in the later stage. From an economic perspective, for mega projects like the one in this paper, if the initial budget allows, the combination of MgO expansion agent and steel fiber is worth promoting, especially in the basement environment of super high-rise buildings, which can effectively reduce the total cost in the later stage of use. But for small projects, if the harm caused by cracking is not serious and easy to repair, then MgO and steel fiber may not be applicable.

## 4. Conclusions

This study addresses the cracking issues in floor concrete and the possible performance deficiencies in the application of MgO expansion agents. The research results indicate that the synergistic effect of steel fibers and MgO expansion agents can effectively improve the mechanical properties of concrete, and the uniform dispersion of fibers is the core factor in improving strength. The main conclusions are as follows:

(1) When steel fiber is used alone, the bond strength between steel fiber and incomplete hydration matrix is low at the early stage, and the fiber–matrix interface transition zone becomes the weak point of concrete, so the fiber can not improve the compressive strength. In the later stage, with the continuous hydration of cement, the matrix–fiber bond strength gradually increased, the fiber gradually played a “bridging effect”, and the compressive strength increased by 7.0%. Without restraint (fiber), harmful expansion will occur when the MgO content reaches 10%, and the strength of M-10-0 is 1.4mpa lower than that of M-8-0, indicating that the MgO content cannot be blindly increased. It is necessary to avoid harmful expansion after excessive MgO under free conditions. In engineering practice, the use of steel fiber and MgO alone has some negative effects;
(2) After using MgO and steel fiber at the same time, the strength of concrete increases by 20.6%, which is greater than the sum of increments when using MgO and steel fiber alone (7.0% + 5.8%), indicating that MgO and steel fiber have a super superposition effect. When the expansion is limited by the steel fiber, it will introduce compressive stress (self-stress) between the fiber and the matrix. This kind of self-stress effectively offsets the shrinkage tensile stress at the initial stage of concrete hardening and reduces the generation of micro-cracks caused by shrinkage. The preloading stress applied by MgO to the matrix can produce a higher bonding force in the interface transition zone, which makes the interface between steel fiber and cement matrix more stable and reduces the occurrence of interface micro-cracks so as to give better play to the reinforcement effect of steel fiber. Of course, this strengthening effect is closely related to the distribution of fibers, and only the uniform distribution of fibers can better restrain the expansion of MgO;
(3) The distribution of fiber has a great influence on the properties of MgO concrete. When the fiber content is small, and the distribution is uneven, it can not play the role of “bridge” and “limiting expansion”. When the fiber content is large, it will produce agglomeration, which has a serious impact on the performance of MgO concrete. Firstly, fiber agglomeration reduces the uniformity of concrete and the bond strength between fiber and matrix. The weak interface between fiber and matrix will become a channel for harmful ions to penetrate, thus reducing the durability of concrete. Secondly, agglomeration reduces the constraint of fiber on MgO expansion and self-stress, which is unfavorable to crack resistance. Finally, due to the increase in fiber content, it also increases the construction cost, resulting in a waste of resources;
(4) When the content of steel fiber is 0.5%, the stability of fiber volume fraction is poor, which is less affected by the content of MgO but more affected by the depth (gravity). When the content of steel fiber is 1.5%, the stability of the fiber volume fraction is very poor. Due to the random fiber agglomeration, the content of steel fiber fluctuates violently. Therefore, in terms of fiber distribution, 1% steel fiber is the most evenly distributed in space, which can better play the role of steel fiber in strengthening and toughening and has a stronger restraining effect on MgO expansion. At this time, the strength of concrete is also higher. When the steel fiber continues to increase to 1.5%, the cost increases, but the strength is no longer significantly improved. It is most reasonable to add 1% steel fiber during construction;
(5) Different from the conventional fiber-reinforced concrete, the uniformity of fiber is more important for the composite system using MgO and steel fiber at the same time. In the composite system, steel fiber not only plays the role of “bridge” but also needs to effectively restrain the expansion of MgO to produce self-stress. Only under the premise of uniform distribution and reliable fiber–matrix bonding can the fiber restrain the expansion. Considering the high price of steel fiber and MgO, adding 8% MgO and 1% steel fiber has the maximum benefit. If the dosage continues to increase, the cost of purchasing raw materials will be significantly increased, but the strength cannot be significantly improved.

## Figures and Tables

**Table 1:** Chemical composition of cement and fly ash/%.

No.	CaO	MgO	Al_2_O_3_	SiO_2_	Fe_2_O_3_	SO_3_	K_2_O	Na_2_O	Loss	Total
Cement	60.9	2.3	6.4	22.4	3.0	1.6	0.58	0.16	2.1	99.4
Fly ash	5.9	1.4	33.9	47.9	6.1	1.4	0.42	0.17	1.9	99.1

**Table 2:** Detailed mix proportions of concrete/kg.

No.	Cement	Fly Ash	Water	Water Reducer	Crushed Stone	Sand	MgO	Steel Fiber
MF-0-0	450	50	160	7	1025	713	0	0
MF-6-0	450	50	160	7	1025	713	30 (6%)	0
MF-8-0	450	50	160	7.5	1025	713	40 (8%)	0
MF 10-0	450	50	160	8.5	1025	713	50 (10%)	0
MF-0-0.5	450	50	160	6	1025	713	0	0.5
MF-0-1	450	50	160	6	1025	713	0	1
MF-0-1.5	450	50	160	6.5	1025	713	0	1.5
MF-6-0.5	450	50	160	7	1025	713	30 (6%)	0.5
MF-6-1	450	50	160	7.5	1025	713	30 (6%)	1
MF-6-1.5	450	50	160	8.5	1025	713	30 (6%)	1.5
MF-8-0.5	450	50	160	7	1025	713	40 (8%)	0.5
MF-8-1	450	50	160	7.5	1025	713	40 (8%)	1
MF-8-1.5	450	50	160	8.5	1025	713	40 (8%)	1.5
MF-10-0.5	450	50	160	7	1025	713	50 (10%)	0.5
MF-10-1	450	50	160	7.5	1025	713	50 (10%)	1
MF-10-1.5	450	50	160	8.5	1025	713	50 (10%)	1.5

**Table 3:** Reinforcement factor of compressive strength at 3 days.

ID	0% Fiber	0.5% Fiber	1% Fiber	1.5% Fiber
0% MgO	0.0%	−5.7%	−2.1%	−3.8%
6% MgO	−0.2%	−4.2%	−2.5%	3.4%
8% MgO	1.7%	1.9%	4.2%	6.1%
10% MgO	−1.1%	4.0%	7.4%	9.3%

**Table 4:** Reinforcement factor of compressive strength at 28 days.

ID	0% Fiber	0.5% Fiber	1% Fiber	1.5% Fiber
0% MgO	0.0%	1.4%	6.1%	7.0%
6% MgO	2.4%	6.5%	12.8%	14.0%
8% MgO	5.8%	15.5%	19.8%	19.1%
10% MgO	3.4%	13.8%	20.6%	17.0%

**Table 5:** Fiber volume fraction of 0% MgO concrete.

Depth	0.5% Fiber	1% Fiber	1.5% Fiber
1	0.36	0.88	1.26
2	0.49	1.14	0.98
3	0.55	0.93	1.73
4	0.62	1.1	2.31
5	0.71	1.15	1.68
Average value	0.55	1.04	1.59
Variation coefficient	0.217	0.108	0.285
Distribution coefficient	0.805	0.897	0.752

**Table 6:** Fiber volume fraction of 6% MgO concrete.

Depth	0.5% Fiber	1% Fiber	1.5% Fiber
1	0.33	1.08	1.56
2	0.35	0.86	1.28
3	0.45	1.06	1.01
4	0.58	1.11	1.68
5	0.71	1.12	2.29
Average value	0.48	1.05	1.56
Variation coefficient	0.297	0.091	0.275
Distribution coefficient	0.743	0.913	0.759

**Table 7:** Fiber volume fraction of 8% MgO concrete.

Depth	0.5% Fiber	1% Fiber	1.5% Fiber
1	0.35	0.89	1.21
2	0.40	1.08	0.93
3	0.42	1.17	1.51
4	0.52	0.98	1.13
5	0.56	1.18	2.13
Average value	0.45	1.06	1.38
Variation coefficient	0.173	0.105	0.302
Distribution coefficient	0.841	0.900	0.739

**Table 8:** Fiber volume fraction of 10% MgO concrete.

Depth	0.5% Fiber	1% Fiber	1.5% Fiber
1	0.39	0.85	1.26
2	0.33	0.83	0.93
3	0.42	0.91	1.28
4	0.56	1.06	1.42
5	0.63	1.02	2.16
Average value	0.47	0.93	1.41
Variation coefficient	0.239	0.098	0.289
Distribution coefficient	0.787	0.907	0.749

## Data Availability

The original contributions presented in the study are included in the article, further inquiries can be directed to the corresponding author.
